# Intracoronary Administration of Microencapsulated HGF in a Reperfused Myocardial Infarction Swine Model

**DOI:** 10.3390/jcdd10020086

**Published:** 2023-02-17

**Authors:** Virginia Blanco-Blázquez, Claudia Báez-Díaz, Francisco Miguel Sánchez-Margallo, Irene González-Bueno, Helena Martín, Rebeca Blázquez, Javier G. Casado, Alejandra Usón, Julia Solares, Itziar Palacios, Rob Steendam, Verónica Crisóstomo

**Affiliations:** 1 Cardiovascular Area, Jesús Usón Minimally Invasive Surgery Centre, 10071 Cáceres, Spain; 2 Centro de Investigación Biomédica En Red de Enfermedades Cardiovasculares CIBERCV, 28029 Madrid, Spain; 3 Stem Cell Therapy Unit, Jesús Usón Minimally Invasive Surgery Centre, 10071 Cáceres, Spain; 4 Immunology Unit, University of Extremadura, 10003 Cáceres, Spain; 5 San Pedro Alcántara Hospital, 10004 Cáceres, Spain; 6 Coretherapix (Tigenix Group, Takeda), 28046 Madrid, Spain; 7 Innocore Pharmaceuticals, 9713 GX Groningen, The Netherlands

**Keywords:** myocardial infarction, swine model, hepatocyte growth factor, microencapsulation

## Abstract

Therapy microencapsulation allows minimally invasive, safe, and effective administration. Hepatocyte growth factor (HGF) has angiogenic, anti-inflammatory, anti-apoptotic, and anti-fibrotic properties. Our objective was to evaluate the cardiac safety and effectiveness of intracoronary (IC) administration of HGF-loaded extended release microspheres in an acute myocardial infarction (AMI) swine model. An IC infusion of 5 × 10^6^ HGF-loaded microspheres (MS+HGF, *n* = 7), 5 × 10^6^ placebo microspheres (MS, *n* = 7), or saline (SAL, *n* = 7) was performed two days after AMI. TIMI flow and Troponin I (TnI) values were assessed pre- and post-treatment. Cardiac function was evaluated with magnetic resonance imaging (cMR) before injection and at 10 weeks. Plasma cytokines were determined to evaluate the inflammatory profile and hearts were subjected to histopathological evaluation. Post-treatment coronary flow was impaired in five animals (MS+HGF and MS group) without significant increases in TnI. One animal (MS group) died during treatment. There were no significant differences between groups in cMR parameters at any time (*p* > 0.05). No statistically significant changes were found between groups neither in cytokines nor in histological analyses. The IC administration of 5 × 10^6^ HGF-loaded-microspheres 48 h post-AMI did not improve cardiac function, nor did it decrease inflammation or cardiac fibrosis in this experimental setting.

## 1. Introduction

Cardiovascular diseases are the leading cause of death in the world [1]. Widespread use of percutaneous coronary angioplasty in the treatment of acute myocardial infarction (AMI) patients has decreased mortality. However, it causes additional damage known as ischemia-reperfusion injury and an adverse ventricular remodeling, increasing the incidence of heart failure [2]. Given the lack of a widely available cure for AMI, it is necessary to develop therapeutic alternatives dealing with the symptoms of the disease and its underlying pathologic processes.

Cell therapy has been one of the main research focuses of AMI treatment, but results of clinical trials have been modest or inconclusive, since these studies have been usually performed on a small patient sample [3].

Current knowledge supports that paracrine signaling is the mechanism by which cell therapy would have a positive effect on damaged tissue. Cells secrete substances directly or encapsulated into vesicles of varied sizes which foster heart repair and protection processes [4,5]. Therefore, new therapeutic strategies based on growth factors administration are being developed to promote cardiac repair after AMI [6].

There is evidence that hepatocyte growth factor (HGF) has cardiac-regenerative properties after stimulating endogenous cardiac stem cells [7,8]. HGF is anti-inflammatory [9] (inhibiting the expression of adhesion molecules and the production of interleukins associated with ventricular remodeling, and releasing anti-inflammatory cytokines) and anti-fibrotic [10] (it increases nitric oxide (NO) in endothelial cells and inhibits TGF-β1 and angiotensin II production). In addition, it reduces oxidative stress [8] (via the activation of PI3K/Akt, MEK/Erk1, 2, p38MAPK and mTOR pathways, and Bcl2 expression, and the inhibition of caspases), and it is angiogenic [11] (through VEGF and IL-8 secretion). Some preclinical studies proved these cardioprotective properties after intramyocardial or intracoronary administration of HGF one month post-AMI [12] or at reperfusion [13]. Based on these studies, small, open-label clinical studies on HGF for ischemic heart disease were performed [14].

The optimal time point for therapy administration in reperfused myocardial infarction treatment is unclear. Some studies defend an early treatment [2], but others prefer a later injection of the therapy after the inflammatory environment has subsided [12]. In our opinion, it should be performed in a balanced moment of the myocardial infarction progress, between the early inflammation and the later fibrosis. Therefore, two days after coronary artery reperfusion could be a perfect moment for the administration of microencapsulated HGF to prevent secondary reactions of myocardial infarction [15,16].

Recently, nanomedicine has advanced in microencapsulation of different therapies that allows their sustained and controlled release to the target area without the need for repeated administrations. Moreover, drug microencapsulation avoids therapies degradation and allows for their minimally invasive administration [17]. In our study, we used biodegradable microspheres (16 µm of diameter) as a vehicle for HGF, which was slowly released over three weeks.

Although previous studies examined the effects of HGF injection [12,13,18], there are no existing experiments that have analyzed their microencapsulated intracoronary administration as a therapy for ischemia-reperfusion injury in a swine model.

Consequently, the main objective of our work was to assess the cardiac safety and effectiveness of an intracoronary (IC) administration of 5 × 10^6^ microspheres containing HGF (60 μg/10^6^ microspheres) at 48 h after reperfused AMI on a porcine model.

## 2. Materials and Methods

**Figure 1:**
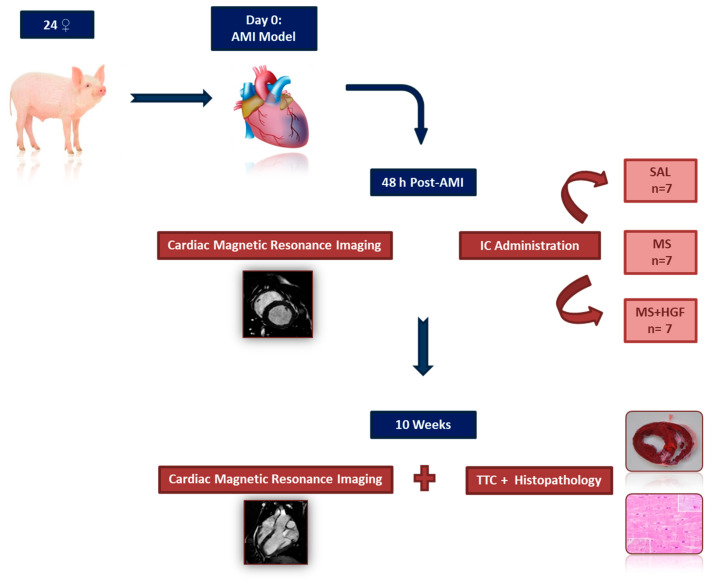
Experimental design. SAL: Saline group; MS: Placebo microspheres group; MS+HGF: Microencapsulated HGF group; AMI: Acute Myocardial Infarction; IC: Intracoronary; TTC: Triphenyltetrazolium chloride.

Once the study protocol was approved by the Ethics Committee of the competent authority (Jesús Usón Minimally Invasive Surgery Centre (JUMISC): Ref.0016/17; Extremadura Regional Government: Exp.20170612), the different phases of the study (Figure 1) were performed according to Spanish Royal Decree 53/2013 and the European regulation (2010/63/EC). A total of 24 female Large White pigs, with an initial mean weight of 32.9 ± 3.6 kg, were used. All animals arrived at the JUMISC at least two weeks before the date of the first intervention, in order to perform clinical examination and allow a quarantine time for detecting the existence of silent pathologies. After the acclimatization period, swine were housed individually in the JUMISC animal housing, where they were fed once a day and allowed ad libitum access to water. All animals received preventive drug therapy (Table 1) for possible thromboembolic processes and/or arrhythmias. The condition of the animals was monitored daily. In the case of signs of heart failure or other complications compromising animal wellbeing, the attending veterinarian criterion as to whether animals were to be excluded from the study (euthanasia) was final.

### 2.1. Anesthesia and Analgesia

Animals were intramuscularly premedicated with diazepam (0.2 mg/kg) and ketamine (10 mg/kg) in order to induce anesthesia later by intravenous (IV) 1% propofol (3 mg/kg). After endotracheal intubation, sevoflurane was used to maintain anesthesia (2–2.5% EtSEVO). Intraoperative analgesia was obtained by an initial IV dose of ketorolac (1 mg/kg) and a continuous remifentanil infusion (15–18 μg/kg/h). Animals also received 2% lidocaine in continuous IV infusion as antiarrhythmic therapy (1 mg/kg/h). They were connected to a semi closed circular anesthetic circuit attached to a ventilator with a fresh gas flow rate of 0.5–0.7 L/min. Ventilation was controlled by a tidal volume of 8–10 mL/kg to obtain normocapnia (35–45 mmHg CO_2_). Breath-holds used in the magnetic resonance studies were performed pausing this anesthetic circuit during image acquisition.

Postoperative analgesia was achieved by intramuscular injection of buprenorphine (10 μg/kg/12 h) during the first day and a fentanyl transdermic release patch (25 μg/h).

### 2.2. Myocardial Infarction Induction

Once premedicated, anesthetized and intubated, the animals were placed in dorsal decubitus on the angio-suite room table with caudal extension of the hindlimbs. Afterwards, the lower-umbilical and groin area was shaved and prepared for surgery in order to allow percutaneous vascular access to a femoral artery (either right or left) by the modified Seldinger technique, using a 7 Fr introducer sheath. After that systemic heparinization with 150 IU/kg of IV sodium heparin was performed and repeated every hour during the intervention.

Selective catheterization of the left coronary artery was carried out under fluoroscopic guidance [19]. A 6 Fr hockey stick guiding catheter was inserted through the 7 Fr femoral sheath over a 0.035” hydrophilic guidewire. The guiding catheter was advanced to the origin of the left coronary artery, and 200 µg of nitroglycerin were administered through the guiding catheter at that point, diluted in 10 mL of 0.9% physiological saline solution as a prophylactic treatment for possible vascular spasms.

A baseline coronary angiography was performed in order to assess coronary flow according to the TIMI scale, and measure the diameter of the paraconal interventricular artery (equivalent to the left anterior descending artery in humans). The diameter of the guiding catheter was used as reference for the measurement. Angiography was performed to image the segment immediately distal to the origin of the first diagonal branch, the target area for angioplasty balloon placement. The contrast medium amidotrizoic acid, diluted to 50% with a saline solution, was manually injected to perform coronariography at the 40° left anterior oblique view (LAO).

**Figure 2:**
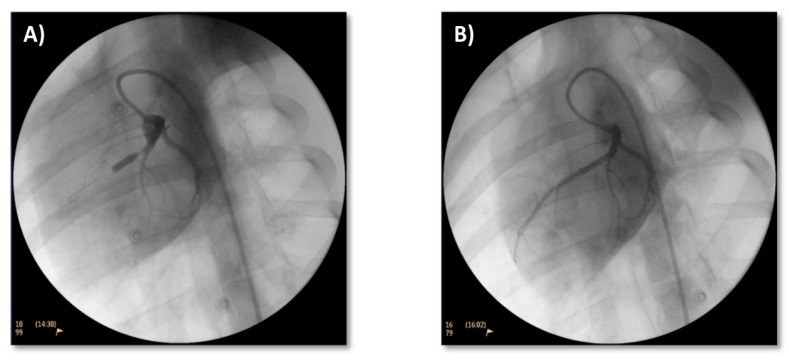
Coronary angiograms. Coronary angiograms in 40° LAO projection to assess proper occlusion of the vessel immediately distal to the first diagonal branch of paraconal interventricular artery (**A**) and vessel patency after removal of angioplasty balloon (**B**).

A 0.014” guidewire was advanced to the distal portion of that artery in order to place over it, in the target area, a coronary angioplasty balloon with a diameter of 2.5–3.5 mm—depending on the target area diameter 1:1.1—and 10 mm in length. A 1 mg/kg 2% lidocaine bolus was administered before coronary occlusion. Balloon insufflation to the nominal pressure was carried out with a manometer, and correct vessel occlusion was checked by coronariography (Figure 2A). Vascular flow interruption was held for 90 min in all subjects. Monitoring of the animals was performed throughout the intervention by electrocardiogram (ECG) in order to identify possible ST-segment changes and to treat arrhythmia or fibrillations with heart massage and/or an external defibrillator, if necessary. Another coronary angiography was carried out in order to assess complete artery occlusion at ischemia after the 90-min period. The balloon was then deflated and removed, and vessel patency checked again by a coronariography (Figure 2B). Finally, the catheter and femoral sheath were removed, and hemostasis was performed by hand compression for 10 min at the arterial access point.

### 2.3. Cardiac Magnetic Resonance Imaging (cMR)

The subjects underwent general anesthesia two days after AMI model induction and 10 weeks after treatment in order to perform cMR (cMR1 and cMR2) examinations. These were carried out by a 1.5 T system (Intera 1.5 T, Philips Medical Systems, Best, The Netherlands) with a Nova gradient system (33 mt/m; 160 mt/m/ms) and a 5-element multi-channel cardiac coil.

Animal hearts were functionally and morphologically assessed, including functional and viability sequences with late enhancement. Images were obtained with cardiac synchronism based on vectocardiogram and at apnea.

Typical parameters used in the cMR examinations were cine sequences with steady-state free precession in order to analyze ventricular function (SENSE × 2, 2.4 ms RT, 1.2 ms ET, 1.6 × 2 mm spatial resolution, 30 phases per cycle, 8 mm slice thickness without gap) and late enhancement of myocardial scar (3D T1 weighted gradient echo, inversion-recovery; pulse delay optimized for maximum elimination of myocardial signal by look-locker sequence; 3.4 ms RT, 1.3 ms ET, 1.4 × 1.7 mm spatial resolution, 5 mm slice thickness, 200–300 ms inversion time).

**Figure 3:**
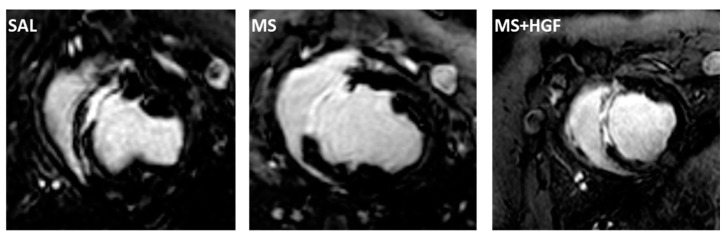
Cardiac Magnetic Resonance. Representative late enhancement images obtained on the short axis view from animals belonging to the three study groups two days after AMI model induction (SAL, MS and MS+HGF).

Cine images and late enhancement were both obtained at the same views: 4 chamber, long axis, and short axis—10–14 consecutive slices, covering both ventricles, from auriculo-ventricular valves to apex—Late enhancement images were obtained 10–15 min following 0.2 mmol/kg gadobutrol administration (Figure 3).

### 2.4. Treatment

Once the cMR1 was performed, the anesthetized swine were taken to the angio-suite room for treatment administration.

The vehicle and means of protection of HGF were the microspheres, which were composed of biodegradable polymers, had a narrow particle size distribution with an average diameter of 16 μm (SynBiosys^®^, InnoCore Pharmaceuticals, Groningen, The Netherlands), and were formulated to allow slow release of HGF at infarction area. Concentration was set at 0.5 × 10^6^ microspheres/mL per dose, to a total dose of 5 × 10^6^ placebo microspheres or HGF-loaded microspheres (60 μg/10^6^ microspheres). This dosage was based on previous studies which determined this optimal dose [16]. HGF is released in vitro over a period of about 6–8 weeks. Thus, the daily release of HGF would be 5 μg/day (60 μg HGF/10^6^ microspheres x 5 × 10^6^ microspheres divided 60 days = 5 μg HGF/day). The vehicle used was 10 mL of physiological saline solution with 5% human serum albumin.

IC administration was performed with a microcatheter at the target area where AMI was induced. 150 μg of nitroglycerin were previously injected to prevent coronary vessel spasms. The administration of nitroglycerin was not performed if the animals presented hypotension, defined as a mean arterial blood pressure below 60 mmHg.

A 2.5 mL solution was given at 1 mL/min followed by a 3-min pause in order to allow treatment diffusion. This injection cycle was repeated four times until dose completion. The volume administered was the same in all groups to ensure blinding. Once the solutions were prepared, they were assigned a code, known only to the authors in charge of preparing the therapies, thus assuring blinding of all the other participants in the study. Three animals died during infarct induction, so surviving subjects were randomly distributed into three study groups: SAL (*n* = 7): 10 mL saline solution; MS (*n* = 7): Placebo microspheres (10 mL; 0.5 × 10^6^ microspheres/mL); MS+HGF (*n* = 7): Microencapsulated HGF (10 mL; 0.5 × 10^6^ microspheres/mL, HGF-loaded with 60 μg/10^6^ microspheres).

A coronary angiogram was performed before and after the treatment to determine the quality of coronary flow.

### 2.5. Blood Tests

**Figure 4:**
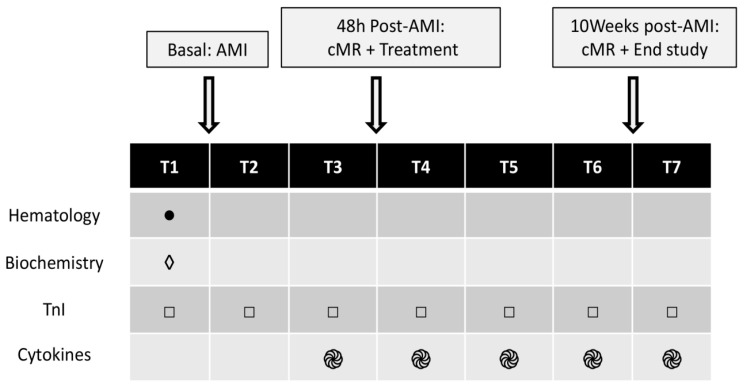
Blood tests. AMI: Acute Myocardial Infarction. Pre- and post-AMI (T1–T2), pre- and post-treatment (T3–T4), 24 h and 1 week post-treatment (T5–T6) and end of study (T7). cMR: Cardiac Magnetic Resonance Imaging. TnI: Troponin I.

Blood tests were performed on all animals at times T1-T7 (Figure 4). Complete hematology and biochemical parameter determinations were carried out at baseline, and troponin I (Tnl) was examined at the remaining time points.

A cytokine panel was assayed (IL-1β, IL-4, IL-6, IL-8, IL-10, IL-12, IFN-α, IFN-γ, TFN-α) from the serum—T3 to T7—of five animals randomly selected from each group (Luminex kit: Cytokine&Chemokine 9-Plex Porcine ProcartaPlex™ Panel 1). The statistical analyses for cytokines were only performed between pre- and post-treatment time points to assess the effect of the therapy on inflammatory parameters.

### 2.6. End of Study: cMR2 and Histology

Ten weeks after IC therapy administration, the last cMR (cMR2), blood test and coronariography were performed. Thereafter, euthanasia was carried out by a lethal dose of potassium chloride (1–2 mmol/kg) while under deep anesthesia. Hearts were harvested and cut into 10–15 mm thick slices. These slices were stained with a triphenyltetrazolium chloride (TTC) solution, in order to macroscopically assess infarct size and site.

The heart samples from infarct and border areas from five animals of each group were histopathologically studied using hematoxylin-eosin (H&E) and Masson’s trichrome with light green (MTC) stain. The histological analyses were performed by a blinded single operator. Inflammation, calcification, necrosis, fibrosis and angiogenesis were graded as described by Fishbein et al. [20]: 0, absent; 1, mild; 2, moderate; and 3, severe, for the histopathologic evaluation of AMI.

### 2.7. Statistical Analysis

The statistics software SPSS 15.0 was used. Data are presented as mean ± standard deviation. The normality test (Shapiro Wilk; *n* < 30) was carried out. Anova tests (comparison of three groups), Student´s *t*-test (two groups) and *t*-test for related samples (intragroups) were used for the variables with a normal distribution. Kruskal-Wallis (3 groups), Mann–Whitney U (2 groups) and Wilcoxon tests (intragroups) were applied for the variables that did not have a normal distribution. Qualitative data were studied using chi-square tests. A *p* < 0.05 level was considered to be significant.

## 3. Results

### 3.1. Model Induction

Three subjects died during AMI induction and were replaced in order to have seven infarcted animals per group. The model was thus created on a total of 24 pigs. Mortality associated with AMI induction was 12.5%.

61.6% of the subjects that survived the model creation suffered from ventricular fibrillation (VF). This type of arrhythmia mostly appeared during coronary vessel occlusion and only in one case during reperfusion.

Baseline estimations of Tnl revealed normal values (0.02 ± 0.02 µg/L). A significant increase (*p* < 0.001; Wilcoxon) was found following reperfusion (9.88 ± 8.06 µg/L), thus confirming myocardial necrosis due to infarction [21].

The animals showed TIMI 3 coronary flow at the coronariography performed to confirm reperfusion, with only one animal showing decreased (TIMI 2) flow.

### 3.2. Treatment

#### 3.2.1. Coronary Flow and ECG

Therapy administration was completed successfully in all animals, except for one (MS group), which died during treatment. This subject started treatment with TIMI 2 coronary flow. Nitroglycerin could not be administered, since the animal suffered from severely low blood pressure. In 29% of cases—all of them from MS and MS+HGF—coronary flow worsened after treatment (Table 2). However, no relevant changes related to the administration were found in electrocardiographic data, except for the case mentioned in MS which suffered from VF and finally died. One animal from MS died during the cMR2 study; therefore, final coronariography could not be performed.

#### 3.2.2. Cardiac Enzymes

A significant decrease in TnI post-administration was observed in the three study groups between T4–T5 and T5–T6 (*p* < 0.05; Wilcoxon) (Table 3). A graphic and a table indicating the individual data of TnI (Figure S1 and Table S1) have been included in the supplementary material to show the distribution of each data group.

#### 3.2.3. Cytokines

IL-4, IL-10, IL-1β, IFN-α, IL-6 and TNF-α values in most animals were under the minimum threshold to be detected by the system. Thus, the corresponding statistical estimations could not be performed.

**Figure 5:**
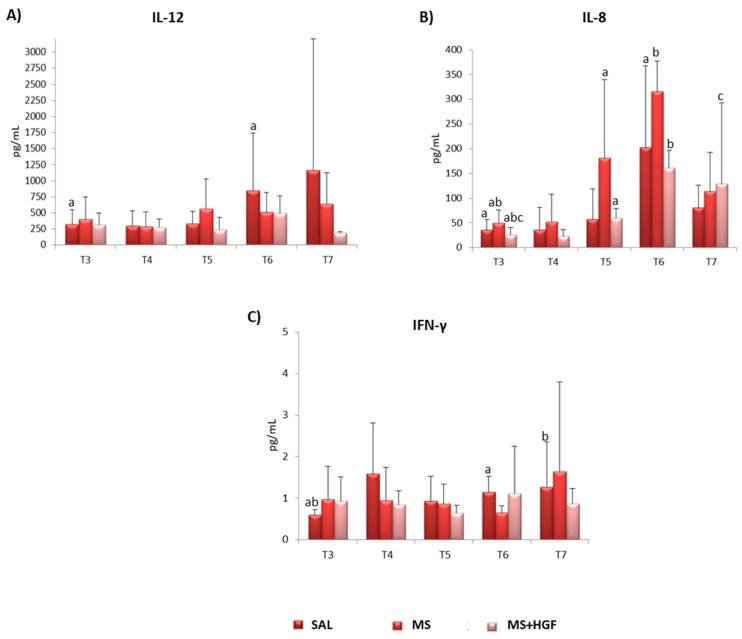
Cytokines: Mean values ± standard deviations of cytokines (pg/mL) from the three groups at T3 (pre-treatment), T4 (post-treatment), T5 (24 h post-treatment), T6 (1 week post-treatment) and T7 (end of study). (**A**) IL-12; (**B**) IL-8; (**C**) IFN-γ. Intragroup differences are determined by ^a,b,c^ *p* < 0.05.

There was only a statistically significant change over time for IL-12 (Figure 5A, Table 4) in SAL group between T3 and T6 (*p* < 0.05; Wilcoxon).

Intragroup significant differences were observed for IL-8 in all groups at various times (*p* = 0.043; Wilcoxon) (Figure 5B, Table 5).

IFN-γ changed significantly in SAL between T3-T6 and T3-T7 (*p* = 0.043 and *p* = 0.042; Wilcoxon (Figure 5C, Table 6).

All the individual data of cytokines are included in supplementary material (Table S2 and Figures S2–S4).

### 3.3. cMR

**Figure 6:**
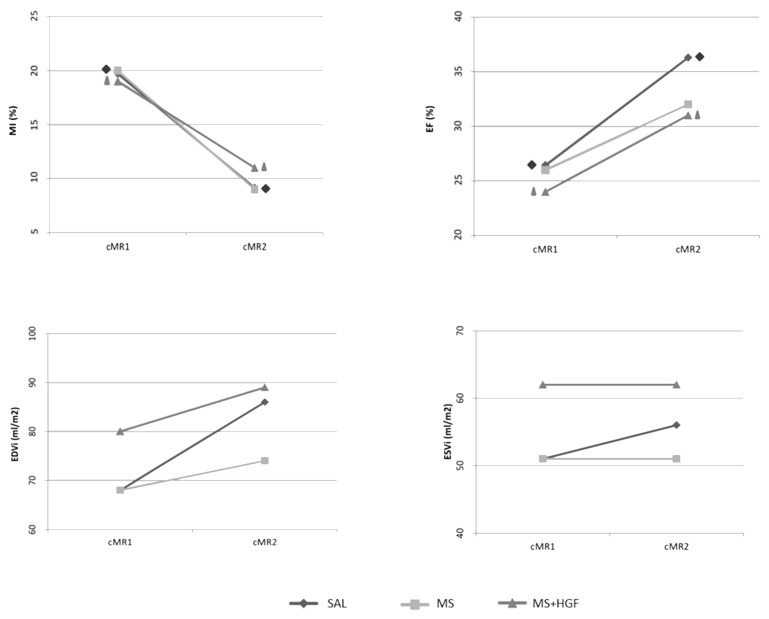
cMR results: cMR1: Pre-treatment; cMR2: End of study; MI: Myocardial infarction percentage; EF: Ejection Fraction; EDVi: End Diastolic Volume (Indexed to Body Surface Area); ESVi: End Systolic Volume (Indexed to Body Surface Area); SAL (*n* = 7); MS (*n* = 7 cMR1, *n* = 5 cMR2); MS+HGF (*n* = 7). Intragroup differences are denoted by Δ, ◊ *p* < 0.05.

One animal died during the second cardiac magnetic resonance examination (cMR2) and was not replaced. cMR data are shown in Table 7. No significant differences in infarct size were found between groups at any study time or in treatment effect (defined as the difference between cMR1 and cMR2 values) (Figure 6). However, a significant decrease in scar size between cMR1 and cMR2 was observed in SAL (*p* = 0.016; Wilcoxon) and MS+HGF (*p* = 0.031; Wilcoxon).

Furthermore, no significant differences were found between the study groups at any time point regarding ejection fraction (EF). A significant increase in this parameter was observed in cMR2 compared to cMR1 in SAL (*p* = 0.031; *t* test for related samples) and MS+HGF (*p* = 0.036; *t* test for related samples) (Table 7).

Individual data of each animal (%MI and % EF) are included in the supplementary material (Figures S5 and S6).

No significant differences between groups or between study time points were found for the ventricular volumes registered in any of the groups (Table 7), although there was a trend towards a limited dilatation in MS and MS+HGF-treated animals compared to SAL.

### 3.4. End of Study

Regarding coronary flow, TIMI 3 was evidenced in 15 and TIMI 2 in four animals—one subject from the SAL, one from the MS and two from the MS+HGF group—at the final coronariography from among all the animals completing the study (*n* = 19). One subject from the MS group died during the second cMR; therefore, this coronariography could not be performed.

**Figure 7:**
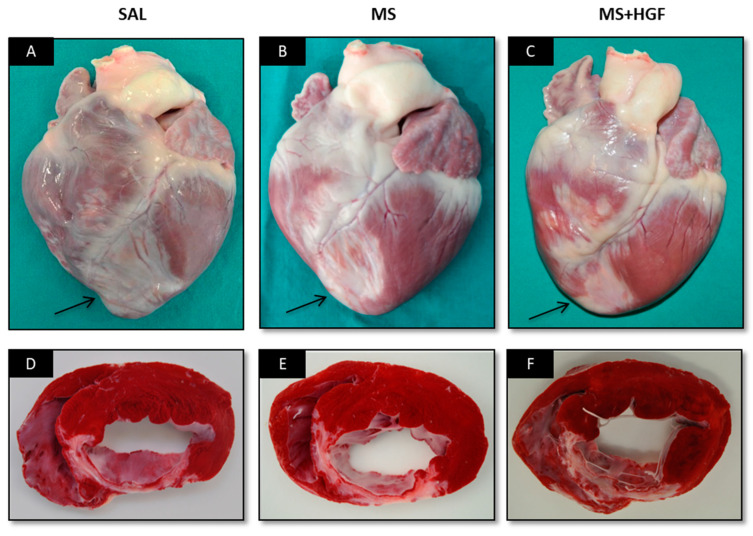
Necropsy study. Photographs of representative hearts from the SAL (**A**,**D**), MS (**B**,**E**) and MS+HGF (**C**,**F**) groups and their TTC-stained slices. The arrows point the infarct area in each group.

No systemic macroscopic changes were found at necropsy study. All hearts showed an anteroseptal and apical whitish area of similar size and thinner than the rest of the myocardium, compatible with infarct area, independently of the group (Figure 7A–C). TTC-stained sections showed anteroseptal infarctions in all cases (Figure 7D–F).

Regarding histopathologic examination, the slices from two animals in the MS group could not be assessed, since they contained several artifacts (MS *n* = 3). No significant differences between groups (*p* > 0.05; Chi-square tests) were found for the histological parameters (Table 8).

## 4. Discussion

At present, the therapies existing for the treatment of AMI patients reduce mortality, but do not deal with the problem of cardiomyocyte loss or damaged tissue regeneration. Therefore, the main goal of our study was to assess IC administration of 5 × 10^6^ microspheres loaded with HGF (60 μg/10^6^ microspheres) at 48 h after inducing AMI on a swine model, under the hypothesis that this could reduce or avoid heart tissue lesions caused by AMI.

Pigs are widely used in cardiovascular research, but they have disadvantages, such as the different coronary artery occlusion level between animals (since the first diagonal branch was not localized at the same place in all of them) and the sensitivity to tachycardia or VF [22] that often results in a high model-related mortality. VF (61.90%) and mortality (12.5%) rates were lower in our study than in other similar models (91% and 30%, respectively) [23,24]. These differences may be due to our using only females—evidence reveals worse survival rates in males, partly because of their greater myocardium thickness [25]—and instituting an amiodarone anti-arrhythmia treatment prior to infarct induction. Our aim was to decrease the defibrillation threshold and the mortality rate compared with other studies [22].

We chose the IC route for our treatment, as this can be performed after revascularization angioplasty and it is a minimally invasive and widely available technique. This allows an easy and safety application of the therapy in clinical practice [26]. Some clinical studies have performed intermittent occlusion for IC administration to improve the retention of the delivered therapy [27,28]. However, experimental studies have demonstrated that retention is not impaired or can even be greater with uninterrupted coronary blood flow during injection [29]. One of the limitations of this route is the risk of increasing the existent microvascular obstruction after infarction if the material to be injected is very large in size. Microspheres composed of biodegradable polymers and with a 16-μm diameter were used in our study. This size is theoretically well tolerated by the heart vessels in pigs and humans [30,31]. Moreover, the Synbiosys^®^ microspheres used in this study are composed of polymers that degraded causing a hydrophilic microenvironment within which the pH of the polymer matrix remains (near) neutral since no acidic degradation products accumulate in the polymer matrix. Preservation of a neutral pH is essential to maintain HGF integrity so it was assumed that bioactivity would be preserved. However, the in vitro study to show the bioactivity after HGF release from microspheres has not been performed. This formulation avoids the passage of microencapsulated growth factors through the coronary capillary bed and into the systemic circulation, until HGF has been unloaded and the particle degraded gradually. Consequently, no traces of these microspheres were found in histopathologic samples at the end of the study.

There are different techniques to assess the microvascular safety of the IC treatment, such as TIMI myocardial perfusion and myocardial blush grade [32,33] but they were not employed in this study. There are prior reports [31] in which microvascular obstruction does not occur after IC infusion of 170 μm encapsulated stem cells, supporting the safety of our approach. In our experiment, we determined coronary flow quality with the TIMI scale and post injection safety by monitoring TnI levels at different study phases. Coronary flow quality after treatment decreased in 5 of the 14 animals that received microspheres (four from the MS group and one from the MS+HGF group). However, this was not accompanied by increased cardiac damage; conversely, there was a significant decrease in TnI post-treatment in treated groups that is compatible with the lack of heart damage [34]. Moreover, since the coronary flow score was TIMI 3 in almost all animals at the end of study, we assume that this compromise in flow was transitory. Furthermore, some authors defend the use of more precise techniques to define coronary physiology, such as measuring the fractional coronary flow reserve (FFR) [30] or the Corrected TIMI frame count [32], since the TIMI scale is subjective and depends on variables such as heart rate and blood pressure, among others [35]. On the other side, as one animal died during therapy and nitroglycerin could not be given to it because of its low blood pressure, patient clinical status should be assessed. IC treatment with these microspheres could be contraindicated for cases presenting adverse events, such as severe low blood pressure or coronary flow change.

There is no consensus as to when the optimal time point is for therapy administration to decrease the inflammation and fibrosis of myocardial infarction. In a prior study using similar drug formulation, the administration at 48 h after infarct induction proved to be safe and effective for improving cardiac function and limiting myocardial fibrosis [17], and therefore the same therapeutic window was used in this study. Furthermore, two days after ischemia-reperfusion injury the inflammation parameters are actually lower than, for example, at post-reperfusion, avoiding a lower effect of the treatment because of the released detrimental metabolites [15].

A decrease in infarct size was observed in all groups at the end of the study in comparison with cMR1, although no significant differences between groups were found. This decrease was statistically significant in SAL and MS+HGF, but not in MS. It could be associated with the reduced number of animals in this group before the cMR2. The decrease in infarct size, frequent in this type of models [19], could be due to an overestimation of infarcted tissue at earlier stages, which is associated with post-infarction inflammation, edema, and hemorrhage. It could also be related to post-AMI ventricular remodeling [36,37,38,39,40]. Both processes led us to choose the functional parameters of ventricular volumes as final outcome measures and EF as heart function predictors, instead of percentage of infarction, as recently described [41].

Ventricular volumes were indexed to body surface area in order to reduce the influence of animal growth on the interpretation of cMR results [19]. A tendency towards an increase in EDVi was found at the end of the study in the three groups, in absence of significant differences between groups or study points. It was similar in ESVi in SAL group but not in MS or MS+HGF group. The criterion for adverse ventricular remodeling, an increase of 20% and 15% in EDVi and ESVi, respectively, was not met in any of the cases [37]. However, this concept is being questioned at present, because it has scarce prognostic value and can hardly be associated with post-AMI cardiovascular events [42,43]. On the other hand, a significant increase in EF and a consequent improvement in heart function were observed in SAL and MS+HGF groups from cMR1 to cMR2, as previously described by Revilla et al. [44]. The absence of significance in MS could be associated with the lower number of animals in this group in cMR2. However, the treatment effect in EF in MS+HGF was not statistically significant compared with the other study groups, so we could not attribute the improvement in heart function to the treatment. Our results differ from those reported by Ellison et al. [13], where a significant improvement of all cMR-analyzed parameters was found in all groups treated with growth factors. This obvious difference in results may be associated with the use of a combination of HGF and IGF-1 in their study, although not all studies using these combinations have reported positive results [45].

Regarding the pro-inflammatory cytokines, a non-significant decrease in IFN-γ and IL-12 was found in the MS+HGF group at the end of the study. This supports the safety of the microspheres delivery, since no increase in these inflammatory markers was seen in animals receiving microspheres, either MS+HGF or MS. Moreover, there could be a certain relation to HGF anti-inflammatory properties, as previously suggested by Rong et al. [8] and the anti-fibrotic role of HGF in ventricular remodeling [9,10,46,47].

The MS+HGF group showed the highest value and a significant increase of the anti-inflammatory cytokine IL-8 at the end of the study. This result agrees with the findings of Frangogiannis et al. [48], who reported IL-8 involvement in neovascularization after infarction. These data could therefore be associated with the pro-angiogenic effect of the therapy analyzed on endothelial cells, since it promotes IL-8 secretion and stabilizes neovessels [11]. However, the absence of significant differences between groups, as well as the increase of this cytokine in the other groups during the study, hinders the interpretation of these results, since they could be associated with the natural recovery of the myocardium after an infarction.

The absence of post-mortem changes (macroscopic examination and final blood tests) could be associated with therapy safety at systemic level. However, biodistribution studies would have had to be performed in order to confirm this, as well as any technique to evaluate the release of growth factor within the myocardium [49]. TTC-stained heart sections revealed anteroseptal and transmural infarctions of similar size in all groups, matching cMR results. Histological results in all groups also showed similar size and transmurality. Conversely, Ellison et al. [13] found more surviving myocardial tissue at the infarcted area of the groups treated with growth factors. These differences could be associated with microsphere use as growth factor carrier in our study and the use of a combination of growth factors in their study. Moreover, the histological parameters showed no significant differences between groups in any case. These results confirm the data obtained from the cMR studies and, unfortunately, the absence of cardiac effectiveness of IC administration of microencapsulated HGF two days after an AMI swine model. However, regarding angiogenesis promotion, necrosis and inflammation, immunohistochemistry techniques should be performed to obtain more information with higher sensitivity of these parameters.

HGF administration was effective and safe in other preclinical studies [9,13,50]. Consequently, there are various clinical trials at present in which patients are recruited for assessment of HGF administration by different routes [51]. Preliminary results of these experimental studies have been positive and support HGF use versus other angiogenic factors. Thus, the study of Wang et al. [11] provided the advances of clinical trials of plasmid and adenovirus HGF in the treatment of critical limb ischemia. It referred the published clinical data which suggest that HGF plasmid was beneficial for improvement of pain and ulcer size in critical limb ischemia patients, although the population was small. Other clinical trials concluded that different routes of administration or doses of HGF plasmid also resulted in an improvement of ulcer healing and reducing rest pain. Moreover, based on the promising preclinical results, intramyocardial injection of adenovirus HGF have been assayed for CHD clinical therapy (Phase I studies). Based on these safety data, a phase II clinical trial was performed in China with adenovirus HGF [52]. This study concluded that the endocardial administration of Ad-HGF improved EF and lowered left ventricular end-diastolic dimension of patients with post-infarct heart failure. Nevertheless, they point out the need to develop release systems for this therapy [11]. While several published works point to a clear benefit of administering HGF in this setting [9,13,50,53], there are also examples of previous studies that obtained unexpected (negative or neutral) results using growth factors therapies, as a recent published paper exposes [45]. In this study, the IC co-administration one week after MI induction of porcine Cardiac Progenitor Cells (pCPC) overexpressing IGF-1 (pCPC-IGF-1) and HGF (pCPC-HGF) did not improve cardiac function. This previous work demonstrated that IGF-1 could have superior potential to promote cardiac differentiation than HGF, as also seen when using encapsulated IGF-1 [17]. It could be associated with a lower stability of the HGF protein compared to the IGF-1 [45]. Therefore, we expected that HGF microencapsulation could provide stability to the growth factor and, as seen with IGF-1, its administration two days after MI induction (between the early inflammation and the later fibrosis) could be effective and would improve cardiac function.

### Study Limitations

One of the main limitations of this study was using young animals, since they were still growing, whereas patients in the clinical environment are usually adults. The results of preclinical studies using young pigs need to be applied carefully in clinical practice, although in this study ventricular volumes are indexed to body surface area in order to minimize the impact of this limitation on cMR results. These animals are young and healthy and do not suffer from any type of associated comorbidity, such as diabetes, hypercholesterolemia, high blood pressure, or from any diet/exercise-associated comorbidity, as is the case in humans [51]. Using adult Large White swine in long-term studies makes handling difficult, because of the large size they reach. Consequently, miniature porcine breeds have been recommended for this type of experiments [54]. In the present study, we have chosen only females because of they have better survival rates than males [25]. However, it could be another limitation since recent guidelines recommend including both sexes in preclinical research [55].

Regarding the coronary physiology, the TIMI scale was the technique used for it, which is subjective and depends on some variables [35]. It could be an important limitation, since the microvascular hemodynamic measurements should be performed using more precise techniques, such as Fractional flow reserve (FFR) or index of microvascular resistance [30]. Moreover, release kinetics of HGF or microspheres were not defined. This issue avoids knowing the bioactivity after HGF release from microspheres.

A further limitation was only using a histological analysis and, therefore, the absence of the immunohistochemistry or oxidative stress pathways parameters that offer more detailed information about angiogenesis, necrosis and inflammation in treated hearts.

## 5. Conclusions

IC administration of 5 × 10^6^ microspheres containing HGF (60 μg/10^6^ microspheres), 48 h after reperfused AMI on a swine model can lead to temporary decrease in coronary flow and does not improve cardiac function. While the results obtained in our study seem not to be promising in this experimental setting, we have only contemplated a single dose and administration time, and therefore cannot say that other studies including different variables (administration routes, doses, injection time point and type of vehicle used) would not be helpful to determine the therapeutic potential of microencapsulated HGF.

## Figures and Tables

**Table 1:** Preventive drug therapy.

DRUG	PRESENTATION	DOSAGE
Amiodarone	200 mg tablets	400 mg/24 h, p. o. From 1 week before AMI and up to 3 days after treatment
Acetylsalicylic acid	500 mg tablets	500 mg/24 h p. o. from one day before AMI until end of study.
Sucralfate	1 g oral suspension	1 sachet/24 h p. o. from beginning to end of study.

p. o.: per os; AMI: Acute Myocardial Infarction.

**Table 2:** Coronary flow evolution at the different study phases.

Group	Pre-Treatment TIMI Flow	Post-Treatment TIMI Flow	End of Study TIMI Flow
SAL	3 (*n* = 2)	3 (*n* = 2)	3 (*n* = 6)
	2 (*n* = 5)	2 (*n* = 5)	2 (*n* = 1)
MS	3 (*n* = 4)	3 (*n* = 1)	3 (*n* = 4)
	2 (*n* = 3)	2 (*n* = 5)	2 (*n* = 1)
MS+HGF	3 (*n* = 3)	3 (*n* = 2)	3 (*n* = 5)
	2 (*n* = 4)	2 (*n* = 4)	2 (*n* = 2)
		1 (*n* = 1)	

**Table 3:** Mean values of TnI.

Group	TnI T3 (μg/L)	TnI T4 (μg/L)	TnI T5 (μg/L)	TnI T6(μg/L)
SAL	11.65 ± 6.45	10.57 ± 5.11 ^a^	7 ± 4.17 ^ab^	0.18 ± 0.24 ^b^
MS	12.97 ± 12.92	7.98 ± 4.03 ^a^	3.59 ± 2.87 ^ab^	0.08 ± 0.06 ^b^
MS+HGF	12.6 ± 5.58	12.43 ± 5.96 ^a^	7.7 ± 3.09 ^ab^	0.17 ± 0.16 ^b^

TnI: Troponin I (μg/L). T3 (pre-treatment), T4 (post-treatment), T5 (24 h post-treatment) and T6 (1 week post-treatment). Data presented as mean ± standard deviation. Intragroup comparisons at different time point are determined by ^a,b,^ *p* < 0.05. SAL (*n* = 7), MS (*n* = 7 T3, *n* = 6 T4,T5,T6), MS+HGF (*n* = 7).

**Table 4:** IL-12. Mean values ± standard deviations of cytokine IL-12 (pg/mL).

Group	T3	T4	T5	T6	T7
SAL	322.1 ± 227.8 ^a^	300.8 ± 235.5	332.6 ± 193.5	845.3 ± 900.3 ^a^	1164 ± 2044.8
MS	403.6 ± 342.9	285.7 ± 230.1	568 ± 460.3	510.5 ± 305.7	644.9 ± 476.4
MS+HGF	312 ± 188.5	272.6 ± 136.8	242.3 ± 185.9	495 ± 266.9	190.8 ± 17.8

Intragroup differences are denoted by ^a^ *p* < 0.05.

**Table 5:** IL-8. Mean values ± standard deviations of cytokine IL-8 (pg/mL).

Group	T3	T4	T5	T6	T7
SAL	35.9 ± 21.6 ^a^	36.2 ± 45.1	57.9 ± 61.1	202.6 ± 164.8 ^a^	80.5 ± 45.5
MS	49.7 ± 25.9 ^ab^	52.6 ± 55.9	181.1 ± 158.6 ^a^	315.5 ± 61.9 ^b^	113.5 ± 79
MS+HGF	25.9 ± 14.6 ^abc^	23.2 ± 14	59.9 ± 19.1 ^a^	160.5 ± 36.6 ^b^	128.5 ± 164.2 ^c^

Intragroup differences are denoted by ^a,b,c^ *p* < 0.05.

**Table 6:** IFN-γ. Mean values ± standard deviations of cytokine IFN-γ (pg/mL).

Group	T3	T4	T5	T6	T7
SAL	0.6 ± 0.1 ^ab^	1.6 ± 1.2	0.9 ± 0.6	1.2 ± 0.4 ^a^	1.3 ± 1.1 ^b^
MS	1 ± 0.8	0.9 ± 0.8	0.9 ± 0.4	0.7 ± 0.2	1.6 ± 2.2
MS+HGF	0.9 ± 0.6	0.8 ± 0.3	0.6 ± 0.2	1.1± 1.1	0.9± 0.4

Intragroup differences are denoted by ^a,b^ *p* < 0.05.

**Table 7:** Cardiac parameters calculated from Magnetic Resonance exams performed through the study.

cMR Parameter	SAL	MS	MS+HGF
%MI cMR1	20 ± 8 ^a^	20 ± 5	19 ± 3 ^a^
%MI cMR2	9 ± 4 ^a^	9 ± 3	11 ± 2 ^a^
∆MI (%)	−11 ± 6	−15 ± 7	−9 ± 4
EF (%) cMR1	26 ± 9 ^a^	26 ± 5	24 ± 8 ^a^
EF (%) cMR2	36 ± 8 ^a^	32 ± 10	31 ± 5 ^a^
∆EF (%)	10 ± 9	6 ±9	7 ±7
EDVi (mL/m^2^) cMR1	68 ± 16	68 ± 10	80 ± 14
EDVi (mL/m^2^) cMR2	86 ± 22	74 ± 13	89 ± 16
∆EDVi (mL/m^2^)	18 ± 27	7 ±13	9 ± 19
ESVi (mL/m^2^) cMR1	51 ± 17	51 ± 9	62 ± 15
ESVi (mL/m^2^) cMR2	56 ± 21	51 ± 12	62 ± 14
∆ESVi (mL/m^2^)	5 ± 26	1 ± 11	0.5 ± 18

cMR: Cardiac Magnetic Resonance imaging; cMR1: Pre-treatment; cMR2: End of study; ∆MI: variation in infarction percentage between cMR1 and cMR2; ∆EF: Ejection Fraction variation between cMR1 and cMR2; ∆EDVi: End Diastolic Volume (Indexed to Body Surface Area) variation between cMR1 and cMR2; ∆ESVi: End Systolic Volume (Indexed to Body Surface Area) variation between cMR1 and cMR2; SAL (*n* = 7); MS (*n* = 7 cMR1, *n* = 5 cMR2); MS+HGF (*n* = 7). Intragroup differences are denoted by ^a^ *p* < 0.05.

**Table 8:** Histological parameters of the three groups with H&E and MTC staining.

Group	Infarction	Necrosis- Apoptosis	Fibrosis	Angiogenesis	Inflammation	Calcification
SAL	1	0	1	1	1	3
	1	0	1	1	2	1
	1	0	1	1	2	1
	1	0	1	1	1	1
	1	0	1	1	1	0
MS	1	0	1	1	1	3
	1	0	1	1	3	1
	1	1	1	1	1	2
MS+HGF	1	0	1	1	1	0
	1	0	1	1	1	0
	1	1	1	1	0	3
	1	0	1	1	1	1
	1	0	1	1	1	0

Scoring of different histopathological parameters: 0, absence of damage; 1, mild moderate severity with small propagation; 2, moderate changes with increase in severity and damage propagation; and 3, severe damage in large part of sample. SAL *n* = 5; MS *n* = 3; MS+HGF *n* = 5.

## Data Availability

The datasets used and/or analyzed during the current study are available from the corresponding author on reasonable request.

## Supplementary Materials

The following supporting information can be downloaded at: https://www.mdpi.com/article/10.3390/jcdd10020086/s1, Figure S1: TnI individual data; Figure S2: Individual data of IFN-g; Figure S3: Individual data of IL-12; Figure S4: Individual data of IL-8; Figure S5: Individual data of %MI; Figure S6: Individual data of %EF; Table S1: Individual TnI data; Table S2: Individual cytokines values.
