# Supplementary material for: Intracoronary Administration of Microencapsulated HGF in a Reperfused Myocardial Infarction Swine Model
Source: J Cardiovasc Dev Dis. 2023 Feb 17;10(2):86. doi: 10.3390/jcdd10020086 (PMC9960949; doi:10.3390/jcdd10020086)
Supplement: Supplementary file 1 [file jcdd-10-00086-s001.zip › jcdd-2101315-supplementary.pdf]

Supplementary material

Table S1: Individual TnI data. The table presents the individual TnI data of each group of the study.

Animal_ID	Group	Time	TnI
P16-361	SAL	1	0.01
P16-747	SAL	1	0.01
P16-744	SAL	1	0.03
P17-015	SAL	1	0.02
P17-359	SAL	1	0.013
P17-360	SAL	1	0.01
P17-369	SAL	1	0.01
P16-361	SAL	2	8.70
P16-747	SAL	2	5.6
P16-744	SAL	2	18.00
P17-015	SAL	2	2.20
P17-359	SAL	2	6.6
P17-360	SAL	2	5.5
P17-369	SAL	2	17
P16-361	SAL	3	4.50
P16-747	SAL	3	7
P16-744	SAL	3	15
P17-015	SAL	3	6.10
P17-359	SAL	3	23
P17-360	SAL	3	12
P17-369	SAL	3	14
P16-361	SAL	4	3.80
P16-747	SAL	4	7.7
P16-744	SAL	4	16.00
P17-015	SAL	4	5.50
P17-359	SAL	4	17
P17-360	SAL	4	11
P17-369	SAL	4	13
P16-361	SAL	5	2
P16-747	SAL	5	6.5
P16-744	SAL	5	11
P17-015	SAL	5	1.10
P17-359	SAL	5	12
P17-360	SAL	5	8.6
P17-369	SAL	5	7.8
P16-361	SAL	6	0.02
P16-747	SAL	6	0.025
P16-744	SAL	6	0.69
P17-015	SAL	6	0.09

P17-359	SAL	6	0.31
P17-360	SAL	6	0.095
P17-369	SAL	6	0.066
P16-361	SAL	7	0.01
P16-747	SAL	7	0.01
P16-744	SAL	7	0.01
P17-015	SAL	7	0.01
P17-359	SAL	7	0.021
P17-360	SAL	7	0.01
P17-369	SAL	7	0.01
P17-018	MS	1	0.02
P17-017	MS	1	0.10
P17-060	MS	1	0.03
P17-058	MS	1	0,03
P17-044	MS	1	0.01
P17-043	MS	1	0.01
P17-046	MS	1	0.01
P17-018	MS	2	34
P17-017	MS	2	13
P17-060	MS	2	8
P17-058	MS	2	2.80
P17-044	MS	2	5.20
P17-043	MS	2	3.70
P17-046	MS	2	20
P17-018	MS	3	9.50
P17-017	MS	3	11
P17-060	MS	3	8.10
P17-058	MS	3	3.50
P17-044	MS	3	14.00
P17-043	MS	3	3,70
P17-046	MS	3	41
P17-018	MS	4	8.90
P17-017	MS	4	9.60
P17-060	MS	4	8.50
P17-058	MS	4	3.40
P17-044	MS	4	14.00
P17-043	MS	4	3.50
P17-046	MS	4	
P17-018	MS	5	5.70
P17-017	MS	5	6.80
P17-060	MS	5	6
P17-058	MS	5	1.70
P17-044	MS	5	0.26
P17-043	MS	5	1.10
P17-046	MS	5	

P17-018	MS	6	0.14
P17-017	MS	6	0.15
P17-060	MS	6	0.12
P17-058	MS	6	0.03
P17-044	MS	6	0.04
P17-043	MS	6	0.02
P17-046	MS	6	
P17-018	MS	7	0.01
P17-017	MS	7	0.02
P17-060	MS	7	0.01
P17-058	MS	7	0.03
P17-044	MS	7	0.14
P17-043	MS	7	0.01
P17-046	MS	7	
P16-367	MS+HGF	1	0.015
P16-444	MS+HGF	1	0.01
P16-552	MS+HGF	1	0.011
P16-553	MS+HGF	1	0.06
P16-551	MS+HGF	1	0.05
P16-734	MS+HGF	1	0.01
P16-733	MS+HGF	1	0.041
P16-367	MS+HGF	2	16
P16-444	MS+HGF	2	3.4
P16-552	MS+HGF	2	4.9
P16-553	MS+HGF	2	16
P16-551	MS+HGF	2	4.90
P16-734	MS+HGF	2	0.06
P16-733	MS+HGF	2	12
P16-367	MS+HGF	3	11
P16-444	MS+HGF	3	6.2
P16-552	MS+HGF	3	17
P16-553	MS+HGF	3	20
P16-551	MS+HGF	3	13
P16-734	MS+HGF	3	5
P16-733	MS+HGF	3	16
P16-367	MS+HGF	4	10
P16-444	MS+HGF	4	5.6
P16-552	MS+HGF	4	11
P16-553	MS+HGF	4	18
P16-551	MS+HGF	4	17
P16-734	MS+HGF	4	5.4
P16-733	MS+HGF	4	20
P16-367	MS+HGF	5	7.6
P16-444	MS+HGF	5	3.5
P16-552	MS+HGF	5	11

P16-553	MS+HGF	5	11
P16-551	MS+HGF	5	8
P16-734	MS+HGF	5	3.7
P16-733	MS+HGF	5	9.1
P16-367	MS+HGF	6	0.024
P16-444	MS+HGF	6	0.025
P16-552	MS+HGF	6	0.33
P16-553	MS+HGF	6	0.45
P16-551	MS+HGF	6	0.10
P16-734	MS+HGF	6	0.07
P16-733	MS+HGF	6	0.21
P16-367	MS+HGF	7	0.01
P16-444	MS+HGF	7	0.01
P16-552	MS+HGF	7	0.01
P16-553	MS+HGF	7	0.01
P16-551	MS+HGF	7	0.01
P16-734	MS+HGF	7	0.01
P16-733	MS+HGF	7	0.012

<u>Figure S1:</u> TnI individual data.

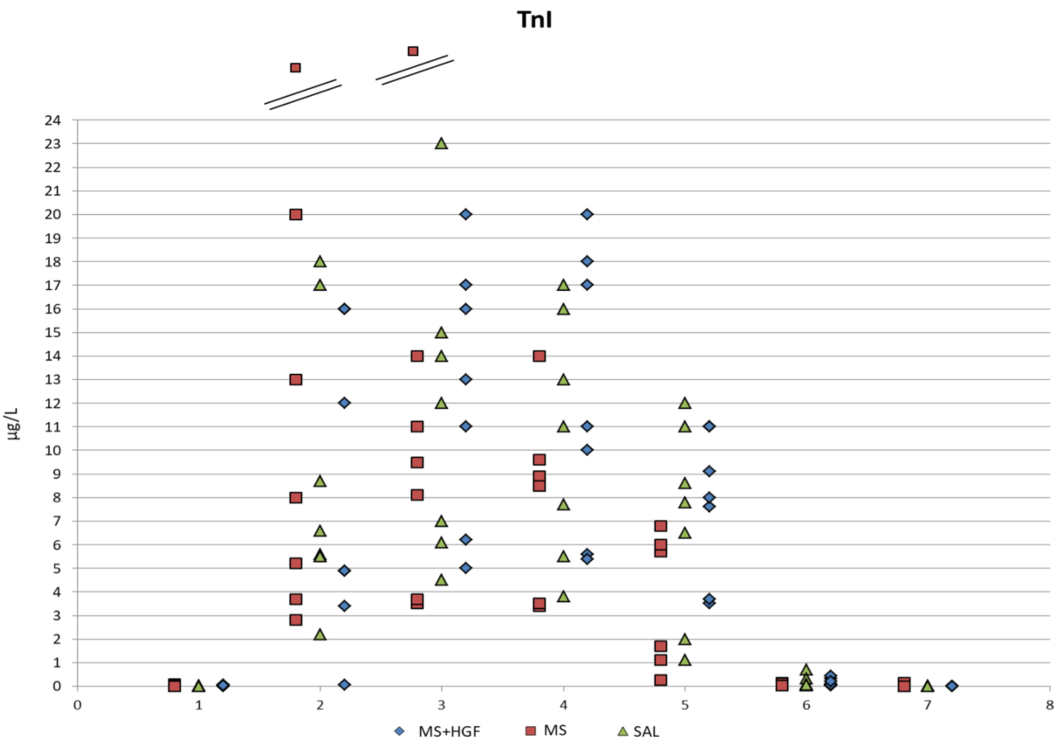

The individual data of TnI of each group have been presented in the graphic along the seven time point of the study. The outside data belong to two animals of the MS group on T2 and T3. The TnI value on T2 is 34 µg/l, on T3 is 41 µg/l. Time point 1 represents data pre- myocardial

infarction model; Time 2: Postmyocardial infarction; Time 3: pre-treatment; Time 4: Post-treatment; Time 5: 24 h post-treatment; Time 6: 1week post-treatment; Time 7: end of study.

<u>Table S2:</u> Individual cytokines values. The table presents the individual data of each cytokine along the study time points

Animal	Time	Group	IFN-g (pg/mL)	IL-12(pg/mL)	IL-8(pg/mL)
1	3	MS+HGF	0.74	353.65	24.71
	4	MS+HGF	0.74	261.6	25.62
	5	MS+HGF	0.95	404.47	68.38
	6	MS+HGF	0.64	523.1	206.68
	7	MS+HGF	1.05	162.62	51.37
2	3	MS+HGF	0.49	95.51	17.04
	4	MS+HGF	0.74	58.37	18.22
	5	MS+HGF	0.64	126	71.8
	6	MS+HGF	0.44	136.12	192.09
	7	MS+HGF	0.53	196.56	420.04
3	3	MS+HGF	1.94	566.26	49.69
	4	MS+HGF	0.64	410.66	45.91
	5	MS+HGF	0.64	98.06	79.77
	6	MS+HGF	0.84	323.85	131.56
	7	MS+HGF	1.27	191.54	89.61
4	3	MS+HGF	0.84	385.89	26.29
	4	MS+HGF	1.44	261.6	9.17
	5	MS+HGF	0.53	100.61	36.04
	6	MS+HGF	3.12	736.18	126.5
	7	MS+HGF	0.44	211.6	41.65
5	3	MS+HGF	0.64	158.84	11.69

	4	MS+HGF	0.64	371.02	17.04
	5	MS+HGF	0.44	482.37	43.36
	6	MS+HGF	0.53	755.86	145.66
	7	MS+HGF	1.05	191.54	39.94
6	3	SAL	0.64	353.65	69.59
	4	SAL	0.64	338.76	36.48
	5	SAL	0.53	305.2	46.75
	6	SAL	0.74	503.36	335.35
	7	SAL	0.84	151.28	46.75
7	3	SAL	0.53	700.5	14.88
	4	SAL	1.05	691.88	16.32
	5	SAL	0.53	593.38	13.42
	6	SAL	1.6	2453.03	88.43
	7	SAL	3.18	4808.78	94.86
8	3	SAL	0.44	118.39	42.93
	4	SAL	3.6	161.36	113.86
	5	SAL	0.44	58.37	25.39
	6	SAL	0.95	472.48	423.82
	7	SAL	0.53	141.18	29.21
9	3	SAL	0.74	201.58	21.72
	4	SAL	1.88	211.6	8.66
	5	SAL	1.55	302.71	39.07
	6	SAL	1.49	437.88	69.99
	7	SAL	0.95	552.7	85.49
10	3	SAL	0.64	236.63	30.33
	4	SAL	0.74	100.61	5.48
	5	SAL	1.6	403.23	164.7

	6	SAL	0.95	359.86	95.44
	7	SAL	0.84	166.4	146.03
11	3	MS	0.64	960.05	67.57
	4	MS	0.44	661.11	31.43
	5	MS	0.84	913.3	145.66
	6	MS	0.53	860.41	253.58
	7	MS	0.84	1363.08	150.81
12	3	MS	0.44	161.36	22.19
	4	MS	0.74	163.88	21.96
	5	MS	0.44	123.46	62.71
	6	MS	0.84	316.39	324.08
	7	MS	0.58	507.06	229.91
13	3	MS	1	92.96	23.34
	4	MS	0.58	49.33	152.28
	5	MS	0.53	9.99	24.25
	6	MS	0.53	98.06	332.33
	7	MS	0.79	181.49	39.07
14	3	MS	2.35	348.69	79.18
	4	MS	2.35	271.58	33.42
	5	MS	0.84	855.49	257.73
	6	MS	0.53	566.26	406.05
	7	MS	5.48	311.42	48.01
15	3	MS	0.44	455.18	56.35
	4	MS	0.58	282.8	23.79
	5	MS	1.66	937.9	415.27
	6	MS	0.84	711.57	261.53
	7	MS	0.53	861.64	99.89

<u>Figure S2:</u> Individual data of IFN-g.

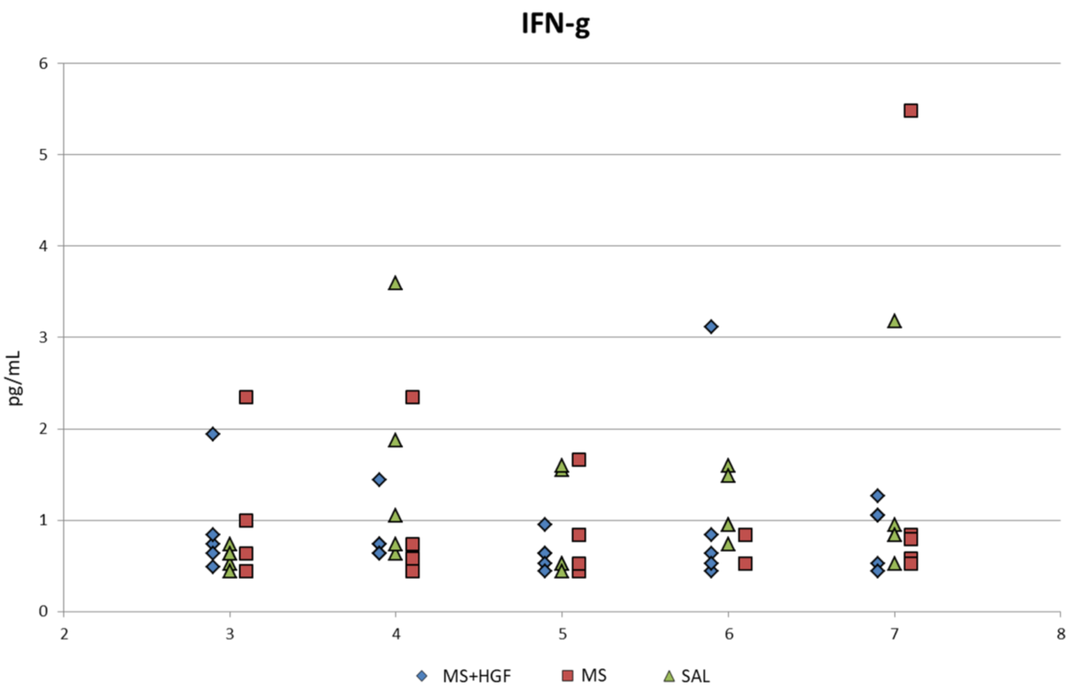

The graphic presents the individual data of IFN-g of each study group along the study time points.

<u>Figure S3:</u> Individual data of IL-12.

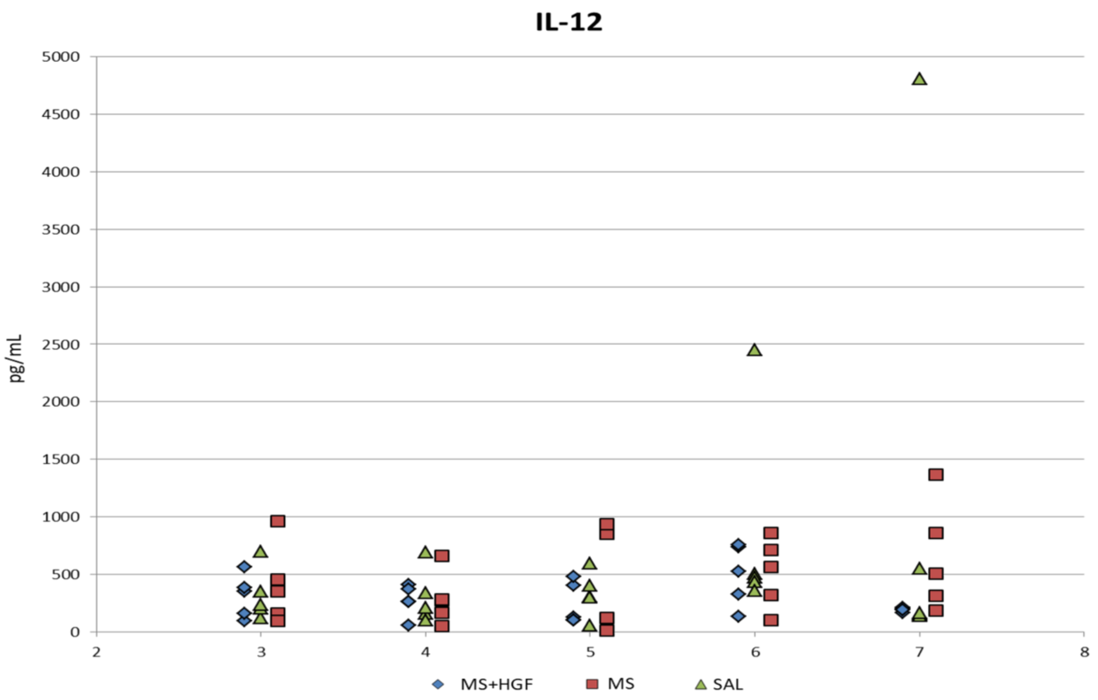

The graphic presents the individual data of IL-12 of each study group along the study time points.

<u>Figure S4:</u> Individual data of IL-8.

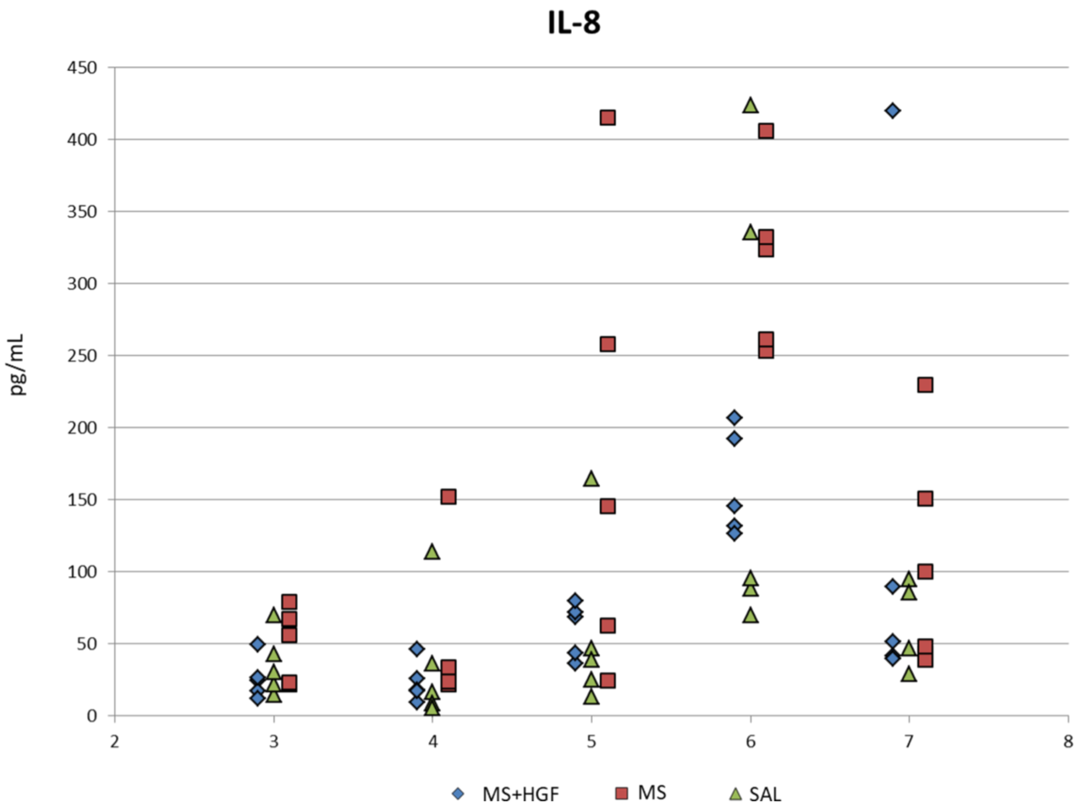

The graphic presents the individual data of IL-8 of each study group along the study time points.

<u>Figure S5:</u> Individual data of %MI

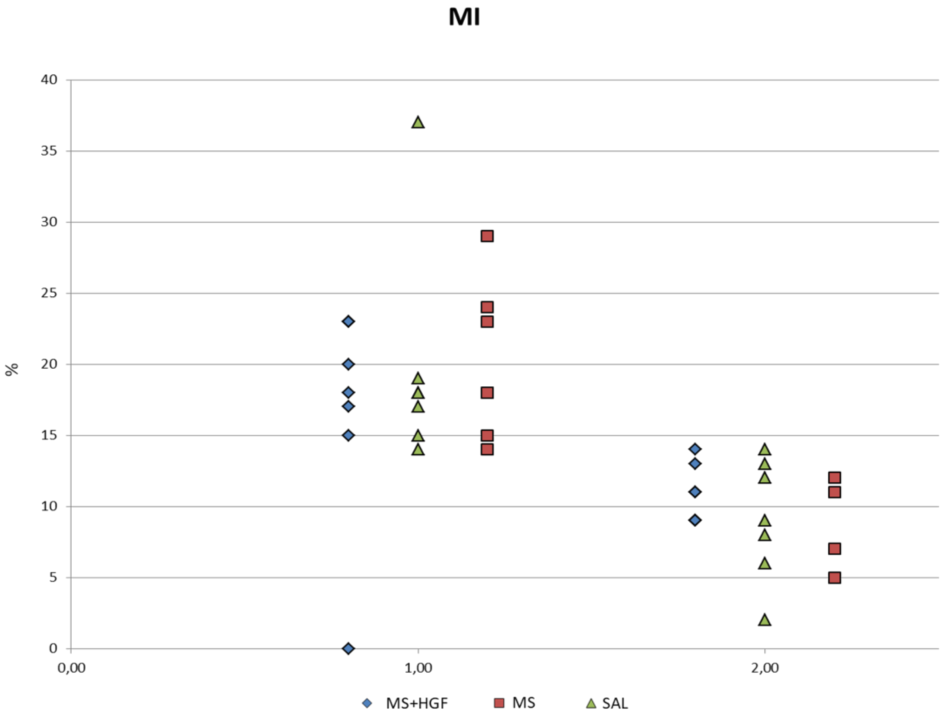

The graphic presents the individual data of % of myocardial infarction measured by Magnetic Resonance of each study group along the study time points.

<u>Figure S6:</u> Individual data of %EF

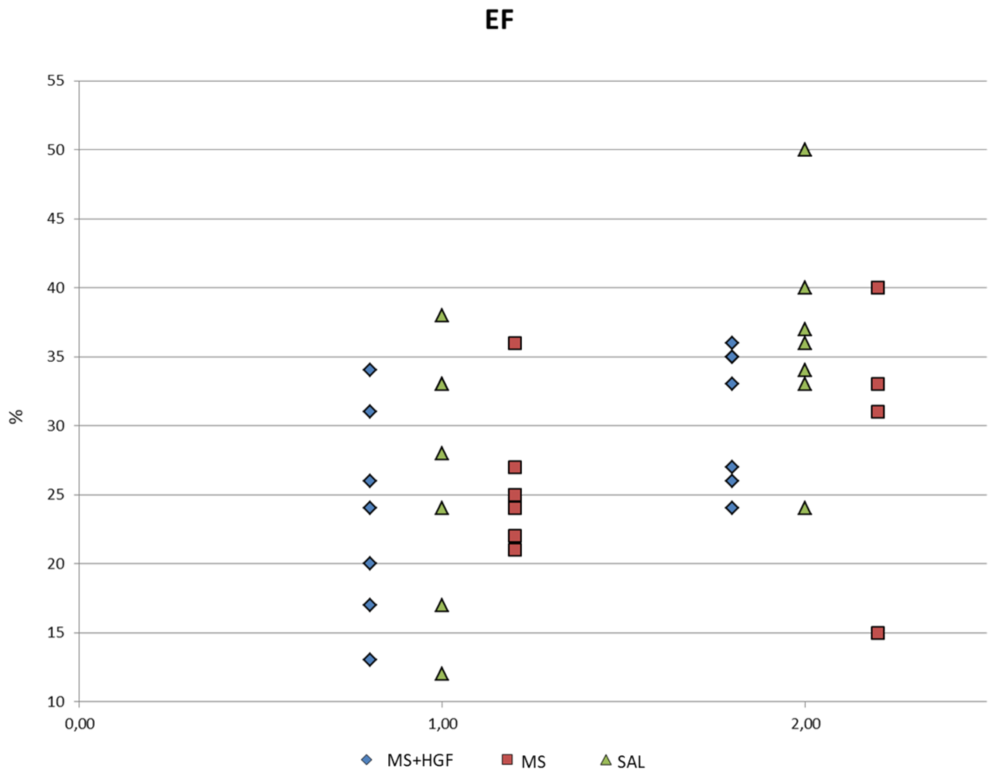

The graphic presents the individual data of % of ejection fraction measured by Magnetic Resonance of each study group along the study time points
